# A Partial Skeleton of the Fossil Great Ape *Hispanopithecus laietanus* from Can Feu and the Mosaic Evolution of Crown-Hominoid Positional Behaviors

**DOI:** 10.1371/journal.pone.0039617

**Published:** 2012-06-25

**Authors:** David M. Alba, Sergio Almécija, Isaac Casanovas-Vilar, Josep M. Méndez, Salvador Moyà-Solà

**Affiliations:** 1 Institut Català de Paleontologia Miquel Crusafont, Universitat Autònoma de Barcelona, Cerdanyola del Vallès, Barcelona, Spain; 2 Department of Vertebrate Paleontology & New York Consortium in Evolutionary Primatology, American Museum of Natural History, New York, New York, United States of America; 3 Institució Catalana de Recerca i Estudis Avançats at Institut Català de Paleontologia Miquel Crusafont and Unitat d’Antropologia Biològica (Departament de Biologia Animal, de Biologia Vegetal i d’Ecologia), Universitat Autònoma de Barcelona, Cerdanyola del Vallès, Barcelona, Spain; Monash University, Australia

## Abstract

The extinct dryopithecine *Hispanopithecus* (Primates: Hominidae), from the Late Miocene of Europe, is the oldest fossil great ape displaying an orthograde body plan coupled with unambiguous suspensory adaptations. On the basis of hand morphology, *Hispanopithecus laietanus* has been considered to primitively retain adaptations to above-branch quadrupedalism–thus displaying a locomotor repertoire unknown among extant or fossil hominoids, which has been considered unlikely by some researchers. Here we describe a partial skeleton of *H. laietanus* from the Vallesian (MN9) locality of Can Feu 1 (Vallès-Penedès Basin, NE Iberian Peninsula), with an estimated age of 10.0-9.7 Ma. It includes dentognathic and postcranial remains of a single, female adult individual, with an estimated body mass of 22–25 kg. The postcranial remains of the rib cage, shoulder girdle and forelimb show a mixture of monkey-like and modern-hominoid-like features. In turn, the proximal morphology of the ulna–most completely preserved in the Can Feu skeleton than among previously-available remains–indicates the possession of an elbow complex suitable for preserving stability along the full range of flexion/extension and enabling a broad range of pronation/supination. Such features, suitable for suspensory behaviors, are however combined with an olecranon morphology that is functionally related to quadrupedalism. Overall, when all the available postcranial evidence for *H. laietanus* is considered, it emerges that this taxon displayed a locomotor repertoire currently unknown among other apes (extant or extinct alike), uniquely combining suspensory-related features with primitively-retained adaptations to above-branch palmigrady. Despite phylogenetic uncertainties, *Hispanopithecus* is invariably considered an extinct member of the great-ape-and-human clade. Therefore, the combination of quadrupedal and suspensory adaptations in this Miocene crown hominoid clearly evidences the mosaic nature of locomotor evolution in the Hominoidea, as well as the impossibility to reconstruct the ancestral locomotor repertoires for crown hominoid subclades on the basis of extant taxa alone.

## Introduction

### The Locomotor Repertoire of *Hispanopithecus Laietanus*

*Hispanopithecus (Hispanopithecus) laietanus* (Primates: Hominidae: Dryopithecinae) is a fossil great ape known from several localities in the Vallès-Penedès Basin (NE Iberian Peninsula) [1]–[9]. For many years, *Hispanopithecus* was treated as a junior subjective synonym of *Dryopithecus* [3], [5]–[7], [10]–[12], but recently it was resurrected [13] for Late Miocene hominids previously lumped into *Dryopithecus*. Two other species are included in the same genus [13]: *Hispanopithecus (H.) crusafonti* [10], [14], also from the Vallès-Penedès Basin; and *H. (Rudapithecus) hungaricus*, from Rudabánya in Hungary [10], [15]–[19]. The latter was previously referred to as *Dryopithecus brancoi* [10], [15]–[17] or *D. carinthiacus* [20], but currently it is designated as *Hispanopithecus hungaricus* [8], [9], [13], [21] (as favored here), or alternatively as *Rudapithecus hungaricus* [18], [19], [22].

The postcranial anatomy of *H. laietanus* is mostly known from the partial skeleton (comprising about 60 elements) from CLL2 [7], [8] (see locality and institutional abbreviations in Table 1), associated with the face from a male adult individual from the same locality [5], [6]. Several features of the thoracic and lumbar vertebrae indicate the possession of a wide and shallow thorax associated with an orthograde body plan [7]. In turn, inferred limb proportions [7], femoral morphology [7], [23], [24] and phalangeal features [7], [8], [25] indicate the possession of adaptations for forelimb-dominated, below-branch suspensory behaviors, including a high intermembral index and long and curved manual phalanges. At the same time, the metacarpal proportions and several morphologic details of the proximal phalanges of *H. laietanus* have been interpreted as indicating the retention of features functionally-related to above-branch quadrupedalism [7], [8], [26]. This has led to the contention that, among fossil crown hominids, palmigrady was gradually abandoned as suspensory behavior became progressively more adaptively significant [8], [9], [25], [26]. Most recently, however, it has been argued that the unusual metacarpo-phalangeal morphology of *H. laietanus* might not reflect the retention of quadrupedal behaviors [22]. Under such view, *Hispanopithecus* would be simply interpreted to display an essentially modern hominoid-like locomotor repertoire, specialized in vertical climbing and suspensory behaviors, but with no significant quadrupedal component. Here we describe a new partial skeleton of *H. laietanus* from Can Feu (CF), which reinforces the contention that this taxon displayed a unique locomotor repertoire combining suspensory and palmigrade behaviors. The significant implications of this assessment for the evolution of crown-hominid positional behaviors are further discussed below.

**Table 1:** Locality and institutional abbreviations.

Abbreviation	Locality or Insitution
ACM	Abocador de Can Mata (Vallès-Penedès Basin, Spain)
AMNH	American Museum of Natural History (New York, USA)
CF	Can Feu (Vallès-Penedès Basin, Spain)
CLL	Can Llobateres (Vallès-Penedès Basin, Spain)
CP	Can Poncic (Vallès-Penedès Basin, Spain)
CV	Can Vila (Vallès-Penedès Basin, Spain)
ICP	Institut Català de Paleontologia Miquel Crusafont (Barcelona, Spain)
IPS	Acronym of the ICP collections
LTR	La Tarumba (Vallès-Penedès Basin, Spain)
TF	Teuleria del Firal (Vallès-Penedès Basin, Spain)

### The *Hispanopithecus* Remains from Can Feu

**Figure 1:**
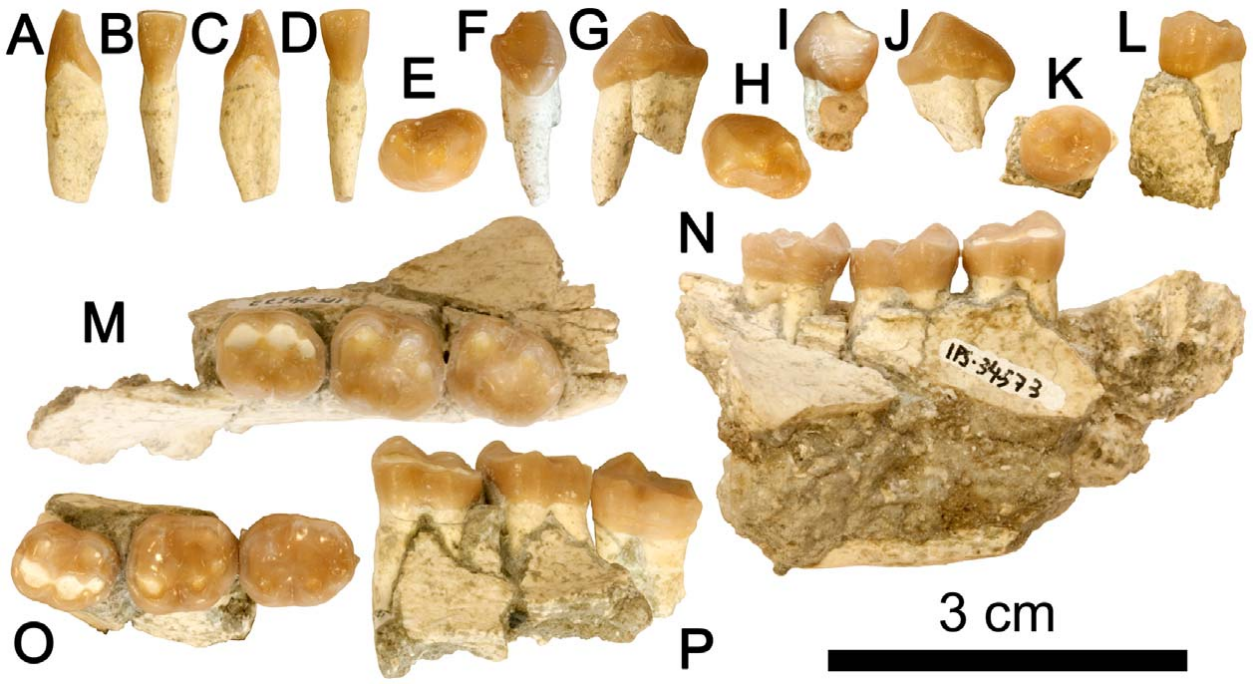
Dentognathic remains of *Hispanopithecus laietanus* IPS34575 from CF1. A–D, Right i1 in mesial (A), lingual (B), distal (C) and labial (D) views; E–G, Left p3 in occlusal (E), mesial (F) and buccal (G) views; H–J, Right p3 in occlusal (H), mesial (I), buccal (J); K–L, Left p4 in occlusal (K) and buccal (L) views; M–N, Mandibular fragment with right m1–m3, in occlusal (M) and buccal (N) views; O–P, Mandibular fragment with left m1–m3, in occlusal (O) and buccal (P) views.

**Figure 2:**
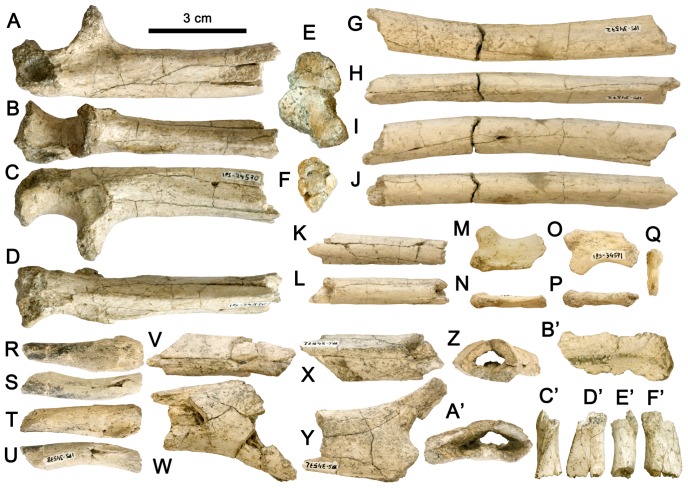
Postcranial remains of *Hispanopithecus laietanus* IPS34575 from CF1. A–F, Proximal fragment of left ulna IPS34575g, in medial (A), anterior (B), lateral (C), posterior (D), proximal (E) and distal (F) views; G–J, Fragments of right radial diaphysis IPS34575h, in lateral (G), anterior (H), medial (I) and posterior (J) views; K–L, Distal fragment of ulnar diaphysis IPS34575j, in lateral (K) and posterior (L) views; M–Q, Proximal fragment of the right first rib IPS34575k, in cranial (M), anterior (N), caudal (O), posterior (P) and proximal (Q) views; R–U, Acromial portion of left clavicle IPS34575l, in cranial (R), posterior (S), caudal (T) and anterior (U) views; V-A’, Distal fragment of left humeral diaphysis, in medial (V), anterior (W), lateral (X), posterior (Y), proximal (Z) and distal (A’) views; B’, Fragment of left scapular blade IPS34575m in posterior view; C’–F’, Lateral fragment of left acromion process IPS34575m, in superior (C’), anterior (D’), inferior (E’) and posterior (F’) views.

**Figure 3:**
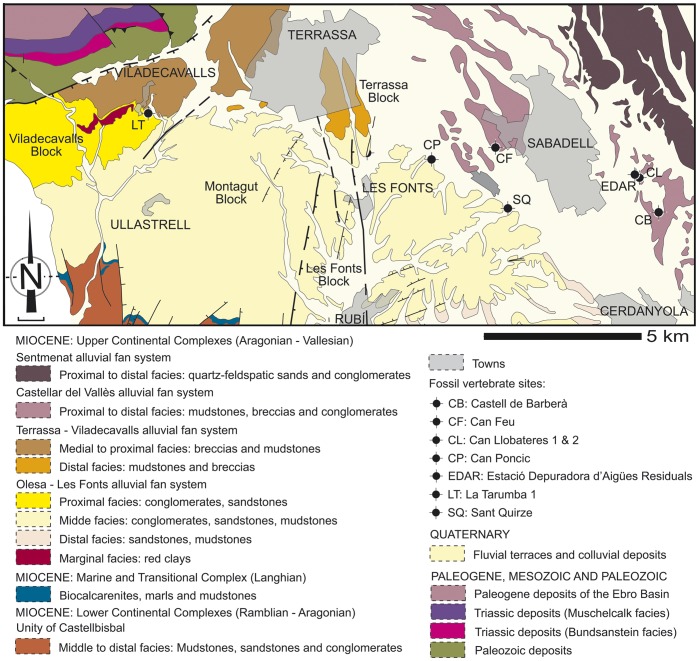
Geologic map showing the situation of selected Vallès-Penedès hominoid localities. Drawn from an original kindly provided by M. Garcés.

The partial skeleton of *H. laietanus* from CF1 (IPS34575; Table 2; Figs. 1, 2) was found in 2001 during the construction of an industrial building at Can Feu [27], [28], which is situated in the Industrial Park of Can Feu (Sant Quirze del Vallès, Catalonia, Spain) [UTM 31T 424185, 4598895], about 4 km E from CLL (Sabadell). Both localities correspond to alluvial plain facies of the Castellar fan system (Fig. 3; Vallès-Penedès Basin) [29], [30]. After the initial discovery, associated sediments were carefully excavated and screen-washed, leading to the recovery of additional remains belonging to a single hominoid individual (IPS34575; see Table 2). The primate skeleton was recovered in a greenish lutite layer (CF1), although most associated micromammal remains come from a blackish lutite layer (CF2) situated 1–2 m above the former [28]. The presence of *Cricetulodon sabadellensis* together with the absence of the murid *Progonomys* enables to correlate CF to the *C. sabadellensis* local range zone of the Vallès-Penedès Basin [27], [28], which ranges from ca. 10.0 to 9.7 Ma (MN9, early Vallesian, Late Miocene) [21]. CF would be therefore contemporaneous or only slightly older than other *Hispanopithecus*-bearing localities from the same area, such as CLL1 (ca. 9.7 Ma) [21].

**Table 2:** Fossil remains of *Hispanopithecus laietanus* IPS34575 from CF1.

Catalogue No.	Description	Figures
IPS34575a	Right mandibular fragment with m1–m3	1R–T, 8A
IPS34575b	Left mandibular fragment with p4 crown and roots	1O–Q, 7A
IPS34575c	Right i1 crown and root	1A–D, 5A
IPS34575d	Left p3 crown and partial roots	1E–I, 6A
IPS34575e	Right p3 crown with partial roots	1J–N, 6B
IPS34575f	Left mandibular fragment with m1–m3	1U–W, 8B
IPS34575g	Proximal fragment of left ulna	2A–F, 9, 10A
IPS34575h	Two diaphyseal fragments of right radius	2G–J
IPS34575i	Distal fragment of left humeral diaphysis	2V-A’
IPS34575j	Distal fragment of ulnar diaphysis	2K–L
IPS34575k	Proximal fragment of right first rib	2M–Q
IPS34575l	Acromial fragment of left clavicle	2R–U
IPS34575m	Two fragments of left scapula	2B’–F’
IPS34575n	Right mandibular condyle and posterior portion of ramus	

## Results

### Body Mass Estimates

The values computed for UTML*  = 14.9 mm, UTSI*  = 17.7 mm and UTDP*  = 10.0 mm, yield a value of UTSA  = 556.27 mm^2^. On the basis of the following allometric prediction equation for extant hominoids [31] ln BM  = 1.314 ln UTSA −5.101, a body mass (BM) estimate of 24.7 kg (50% CI 22.8–26.8 kg) is obtained. With regard to radial diameters, the measurements of R50ML  = 9.2 mm and R50AP  = 11.4 mm yield a value of R50AB  = 10.3 mm. Based on the allometric prediction equation for extant hominoids [31] ln BM  = 2.798 ln R50AB –3.416, a BM estimate of 22.0 kg (50% CI 19.5–24.9 kg) is obtained, being thus only slightly smaller than the estimate obtained from ulnar articular measurements. A BM around 22–25 kg can be therefore inferred for the CF partial skeleton. This BM estimate agrees well with the female sex inferred on the basis of p3 size and morphology (see below), being lower than the 39 kg (50% CI 34–43 kg) estimated for the male skeleton IPS18800 from CLL [7] on the basis of femoral head dimensions [13]. This suggests that *H. laietanus* displayed a significant degree of body size dimorphism (males about 50% larger than females), as it is common in Miocene and extant great apes [32], being intermediate between the moderate dimorphism displayed by chimpanzees and bonobos (about one-third larger) and the higher dimorphism displayed by gorillas and orang-utans (more than twice as heavy) [33].

### Description of Dental Morphology

**Figure 4:**
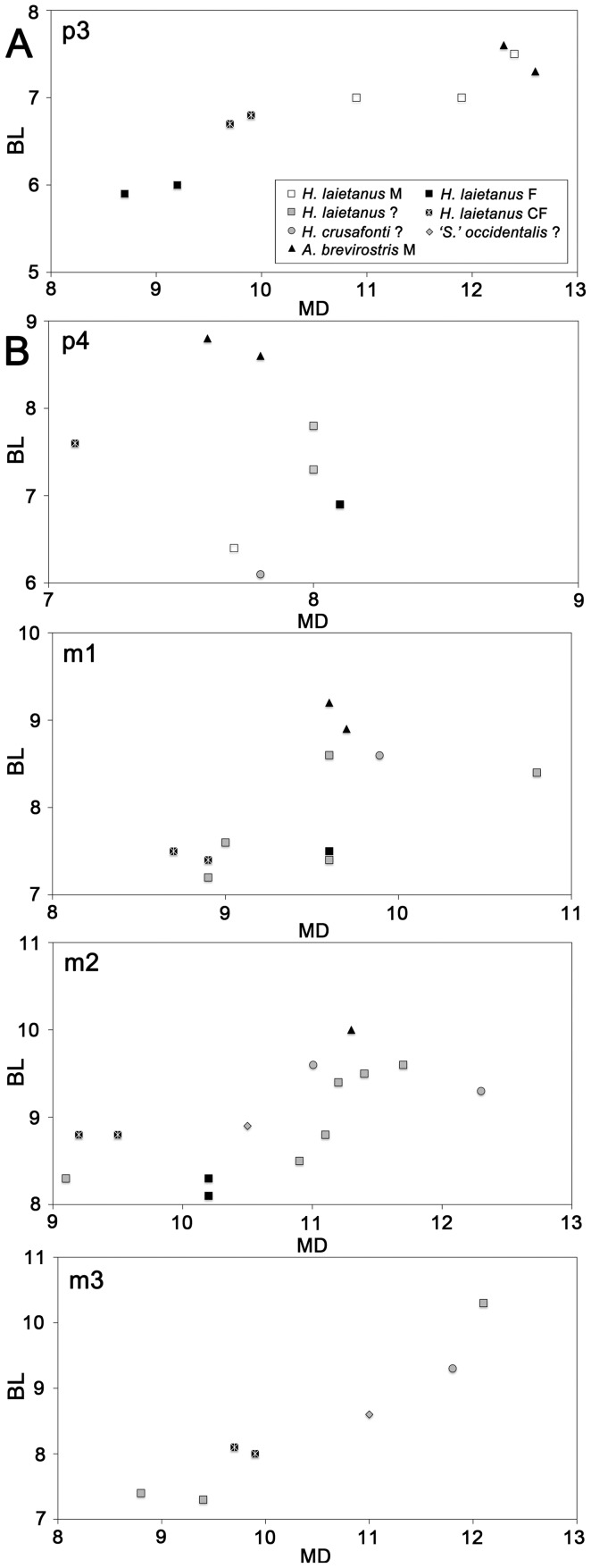
Lower cheek-teeth proportions of Vallès-Penedès hominoids. The depicted taxa included *H. laietanus* (CF1, CLL1 and LTR1), *H. crusafonti* (CP and TF), *Anoiapithecus brevirostris* (ACM/C3-Aj) and *‘Sivapithecus occidentalis’* nomen dubium (CV). All measurements were taken by the senior author of this paper (DMA). A, p3; B, p4; C, m1; D, m2; E, m3.

**Figure 5:**
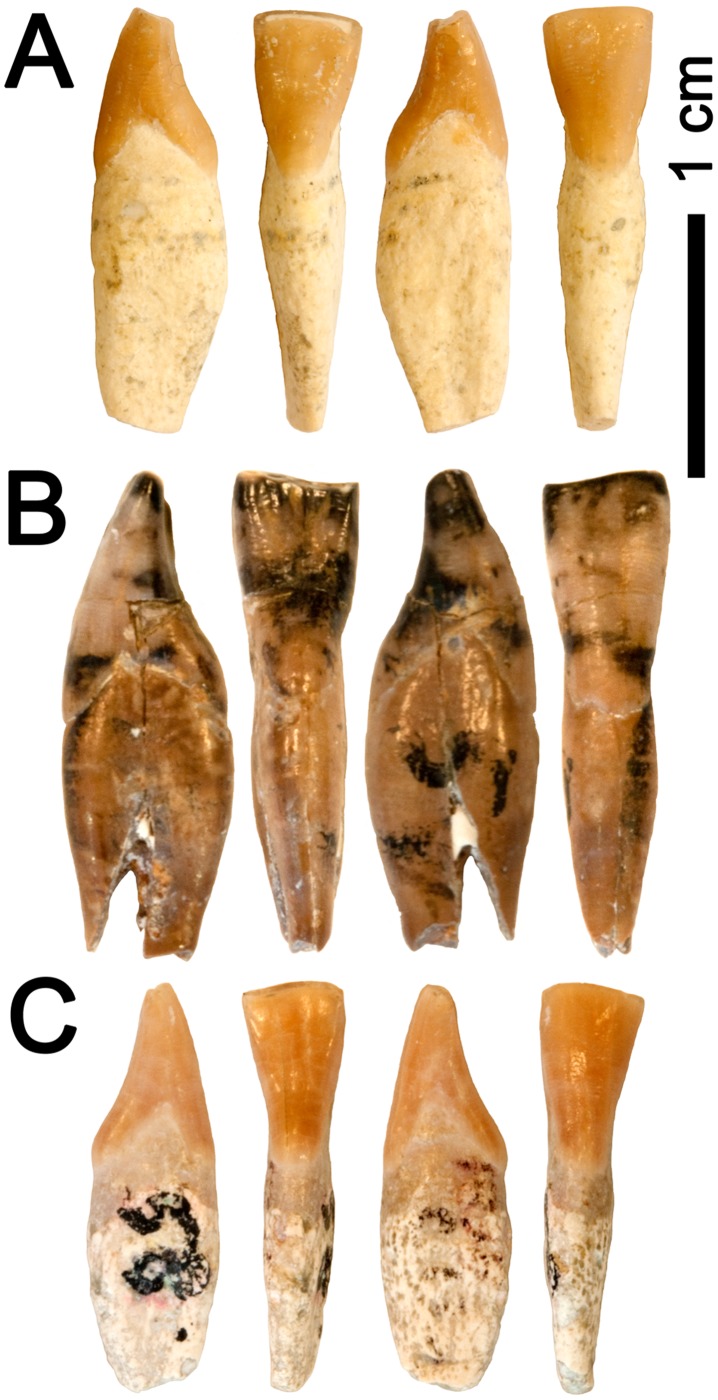
Lower incisors of *Hispanopithecus laietanus*. Each specimen depicted (from left to right) in mesial, lingual, distal and labial views. A, Right i1 IPS34575c from CF1; B, Right i1 IPS1841 from CLL1; C, Left i2 IPS1838 from CLL1.

**Figure 6:**
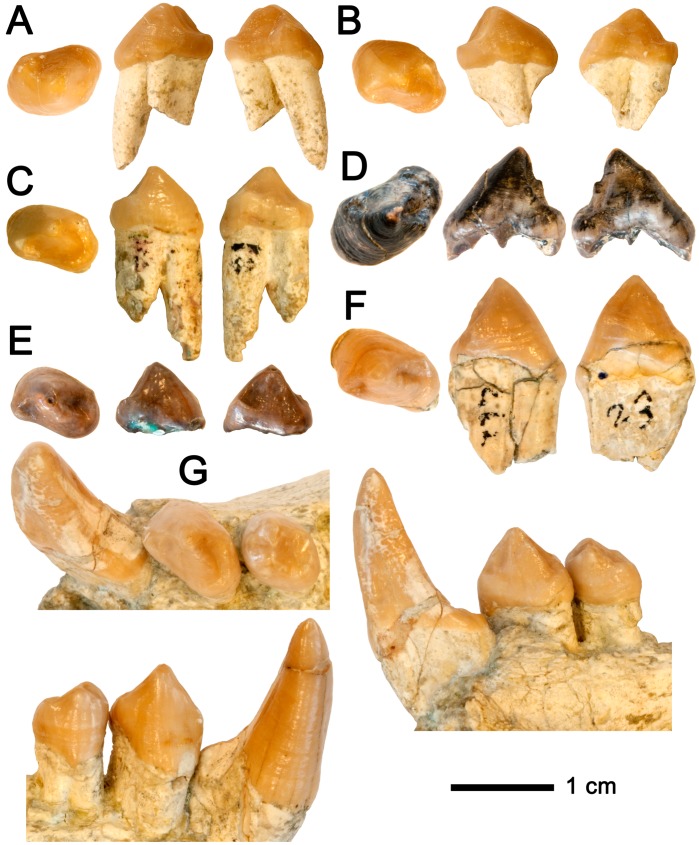
Lower third premolars of *Hispanopithecus laietanus*. Each specimen depicted (from left to right) in occlusal, buccal and lingual views. A, Female left p3 IPS34575d from CF1; B, Female right p3 IPS34575e from CF1; C, Female right p3 IPS1762 from CLL1; D, Male left p3 IPS1791 from CLL1; E, Female right p3 IPS1803 (holotype) from LTR1; F, Male right p3 IPS1777 from CLL1; G, Male right c1-p4 IPS1764 from CLL1.

**Figure 7:**
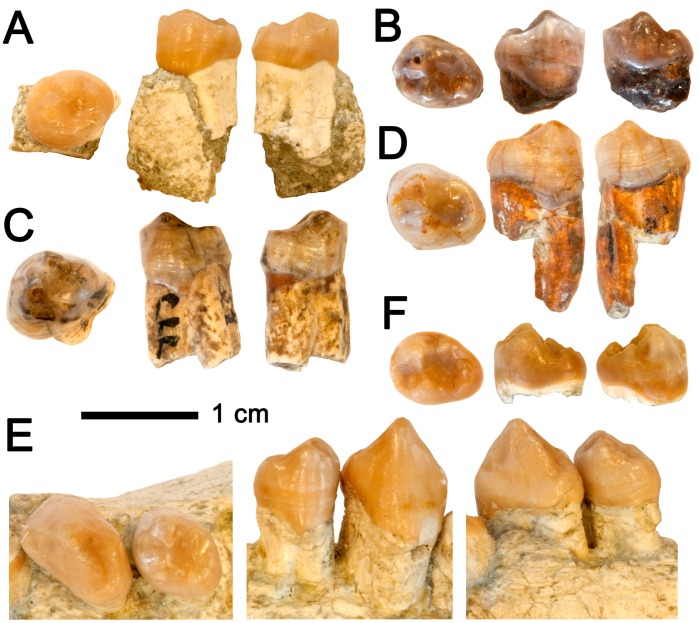
Lower fourth premolars of *Hispanopithecus* spp. Each specimen depicted (from left to right) in occlusal, buccal and lingual views. A, Female left p4 IPS34575b of *H. laietanus* from CF1; B, Female right p4 IPS1803 of *H. laietanus* (holotype) from LTR1; C, Left p4 IPS1775 of *H. laietanus* from CLL1; D, Right p4 IPS1776 of *H. laietanus* from CLL1; E, Male right p1–p4 IPS1764 from CLL1; F, Right p4 IPS1811 of *H. crusafonti* from CP.

Detailed descriptions are reported in the Text S1, so that only comparative descriptions are provided below. The lower central incisor and the lower cheek teeth are preserved (Fig. 1; Table 2; see Table 3 for measurements, Fig. 4 for proportions, and Figs. 5, 6, and 7 for comparison with other *Hispanopithecus* specimens). The i1 (Figs. 1A–D, 5A) is a spatulate and waisted tooth, similar but smaller than the i1 from CLL1 (Fig. 5B) [2], [3], [34]. Both specimens display a longer and more symmetrical crown than an i2 from CLL1 (Fig. 5C), alternatively interpreted as a di1 [3] or i1 [34]. The p3 (Figs. 1E–N, 6A–B) is sectorial and displays a wide mesiobuccal honing facet, metrically and morphologically resembling the holotype from LTR1 (Fig. 6E) [1], [2] and another *H. laietanus* specimen from CLL1 (Fig. 6C) [2], attributed to female individuals [2]. These specimens differ from male p3 from CLL1 (Figs. 6D,F,G) [2], [34] in their lower and less elongated crown (Fig. 4A) and the less fused mesial and distal roots. The p4 (Figs. 1O–Q, 7A) displays a suboval profile and resembles both the holotype (Fig. 7B) [1], [2] and other *H. laietanus* specimens from CLL1 (Figs. 7C–E) [2], although being somewhat shorter and relatively broader (Fig. 4B). The only p4 of *H. crusafonti* from CP (Fig. 7F) [2], [14] is more buccolingually-compressed (Fig. 4B), with a more elongated and tapering talonid. In contrast, the p4 of *Anoiapithecus* [35] is absolutely and relatively broader (Fig. 4B), and displays a less restricted mesial fovea.

**Table 3:** Dental measurements of *Hispanopithecus laietanus* from CF1.

Catalogue No.	Tooth	MD	BLm	BLd
IPS34575a	Rm1	8.9	7.3	7.4
IPS34575a	Rm2	9.5	8.8	8.3
IPS34575a	Rm3	9.7	8.1	7.7
IPS34575f	Lm1	8.7	7.5	7.5
IPS34575f	Lm2	9.2	8.8	8.2
IPS34575f	Lm3	9.9	8	7.6
IPS34575c	Ri1	4.2	4.8	
IPS34575d	Lp3	9.7	6.7	
IPS34575e	Rp3	9.9	6.8	
IPS34575b	Lp4	7.1	7.6	

Abbreviations: R, right; L, left; MD, maximum mesiodistal length; BLm, maximum buccolingual breadth in premolars, and breadth of the mesial lobe in molars; BLd breadth of the distal lobe in molars.

**Figure 8:**
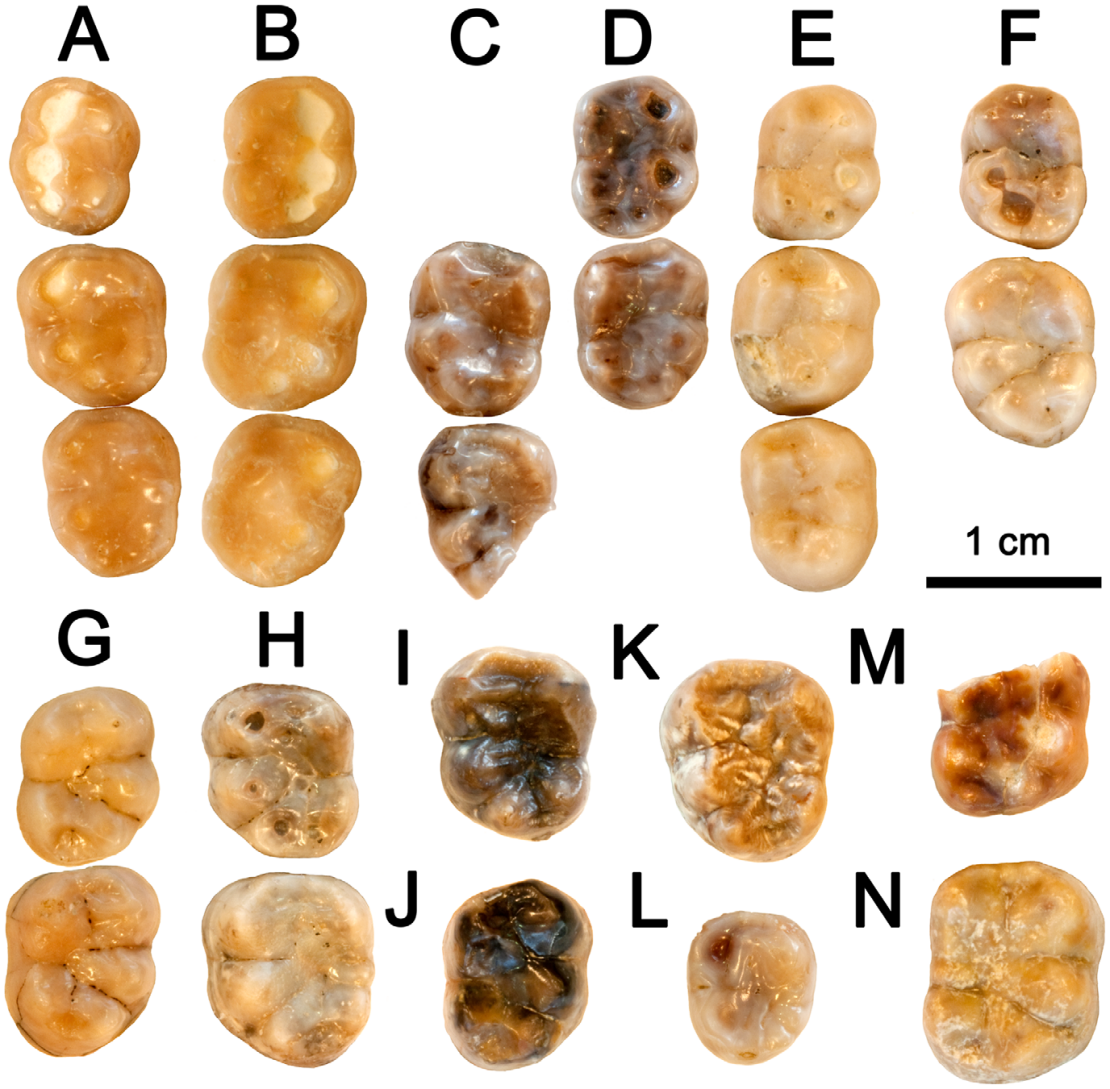
Lower molars of *Hispanopithecus* spp. All specimens depicted in occlusal view. A, Female right m1–m3 IPS34575f of *H. laietanus* from CF1; B, Female right m1–m3 IPS34575a of *H. laietanus* from CF1; C, Left m2–m3 IPS1804 (holotype) of *H. laietanus* from LTR1; D, Right m1–m2 IPS1803 (holotype) of *H. laietanus* from LTR1; E, Right m1–m3 IPS1802 of *H. laietanus* from CLL1; F, Left m1–m2 IPS1796 of *H. laietanus* from CLL1; G, Right m1–m2 IPS1797 of *H. laietanus* from CLL1; H, Left m1–m2 IPS9001 of *H. laietanus* from CLL1; I, Left m2 IPS1782 of *H. laietanus* from CLL1; J, Right m2 IPS1780 of *H. laietanus* from CLL1; K, Left m3 IPS1822 of *H. laietanus* from CLL1; L, Left m3 IPS1800 of *H. laietanus* from CLL1; M, Right m1 IPS1813 of *H. crusafonti* from CP; N, Right m2 IPS1816 of *H. crusafonti* from CP.

The lower molars (Figs. 1R–W, 8A–B) are subrectangular and display a Y5 occlusal pattern, with a short mesial fovea, a more extensive talonid basin, and a restricted and lingually-situated distal fovea; there are no cingulids, and the lingual cuspids are more peripheralized than the buccal ones, with the hypoconulid situated buccally but close to crown midline. The CF molars resemble in size, proportions (Figs. 4C–E) and occlusal morphology the holotype (Figs. 8C–D) and other *H. laietanus* specimens from CLL1 (Figs. 8E–L), although the latter (particularly the m3; Figs. 4E, 8A–C,E,K–L) show some degree of intraspecific variability in morphology and proportions. The CF specimens are close to the lower size range of *H. laietanus* (Figs. 4C–E), and they all differ from *H. crusafonti* from CP (Figs. 8M–N) and TF [14] by the less quadrangular occlusal profile and more extensive talonid basin. The longer postmetacristid and longer pre-entocristid in the only complete CP lower molar (Fig. 8N) is too variable to be a reliable diagnostic criterion [11], like the presence of a distinct metaconulid in the former (since it is also present in some CLL1 specimens; Figs. 7A–B, H). Like other *Hispanopithecus* specimens, the CF m1 and m2 differ from those of *Anoiapithecus* in the relatively narrower crown (Figs. 4C–D), the narrower buccal cuspulids, the less centrally-placed hypoconulid, and the lack of cingulids.

### Description of Postcranial Remains

Several postcranial bones of the shoulder girdle, rib cage and forelimb are preserved (Table 2; Fig. 2; see Supplementary Information for more detailed descriptions). The former include two scapular fragments (Figs. 2B’–F’) and the acromial end of the clavicle (Figs. 2R–U), which were previously unknown for *Hispanopithecus*–the acromial end is not preserved in the purported clavicular fragment from the CLL2 male individual of *H. laietanus* [7]. The scapular spine (Fig. 2B’) is straighter than in extant hominoids, suggesting a different (more monkey-like) shape of the scapular blade, whereas the acromial fragment (Figs. 2C’–F’) indicates a longer and more compressed acromion process than in monkeys (somewhat derived towards the hominoid condition).

The clavicular fragment (Figs. 2R–U) is very straight, differing from extant hominoids (which display a marked sigmoid curvature) and even monkeys (which display a well-defined curvature of the acromial end). Early and Middle Miocene apes (*Proconsul*, *Equatorius*, *Nacholapithecus* and *Pierolapithecus*) display a robust clavicle with a faint sigmoid curvature [36]–[37], similar to that of colobines [37], thus being less curved and displaying less marked muscular insertions than in extant apes [39], [40]. Among fossil apes, the CF specimen most closely resembles the partial clavicle of *Equatorius*, although given its incompleteness functional inferences are precluded. From the rib cage, only a first rib proximal portion (Figs. 2M–Q) is preserved. Although no comparisons with fossil apes can be provided, it displays a mix of characters, with a protuberant tubercle as in monkeys, hylobatids and humans, a neck-shaft angle similar to hylobatids and extant hominines (lower than in monkeys and orangutans), and a craniocaudally-compressed shaft (as in extant apes), further lacking the proximal shaft constriction displayed by monkeys.

Among the forelimb remains, the humeral fragments (Figs. 2V-A’) do not enable well-founded comparisons (Fig. S1). However, the marked lateral supracondylar crest, the flattened distal shaft and the wide shaft portion lateral to the olecranon fossa suggest a modern hominoid-like distal humeral morphology, more derived than in *Proconsul*, and more similar to that of kenyapithecines (such as *Nacholapithecus*), *Sivapithecus* and, especially, *Dryopithecus fontani* (Figs. S1B–C) [41]–[43] and *H. hungaricus* [42], [44]. The preserved radial diaphysis (Fig. 2G–J) is smaller and more slender than the male specimen from CLL2 [7], representing about the same shaft portion. Both display a similar mediolaterally-compressed outline, which differs from the rounder profile displayed by extant hominoids and rather resembles quadrupedal monkeys. The distal fragment of ulnar diaphysis (Figs. 2K–L) is not very informative, unlike the proximal partial ulna (Figs. 2A–F).

**Figure 9:**
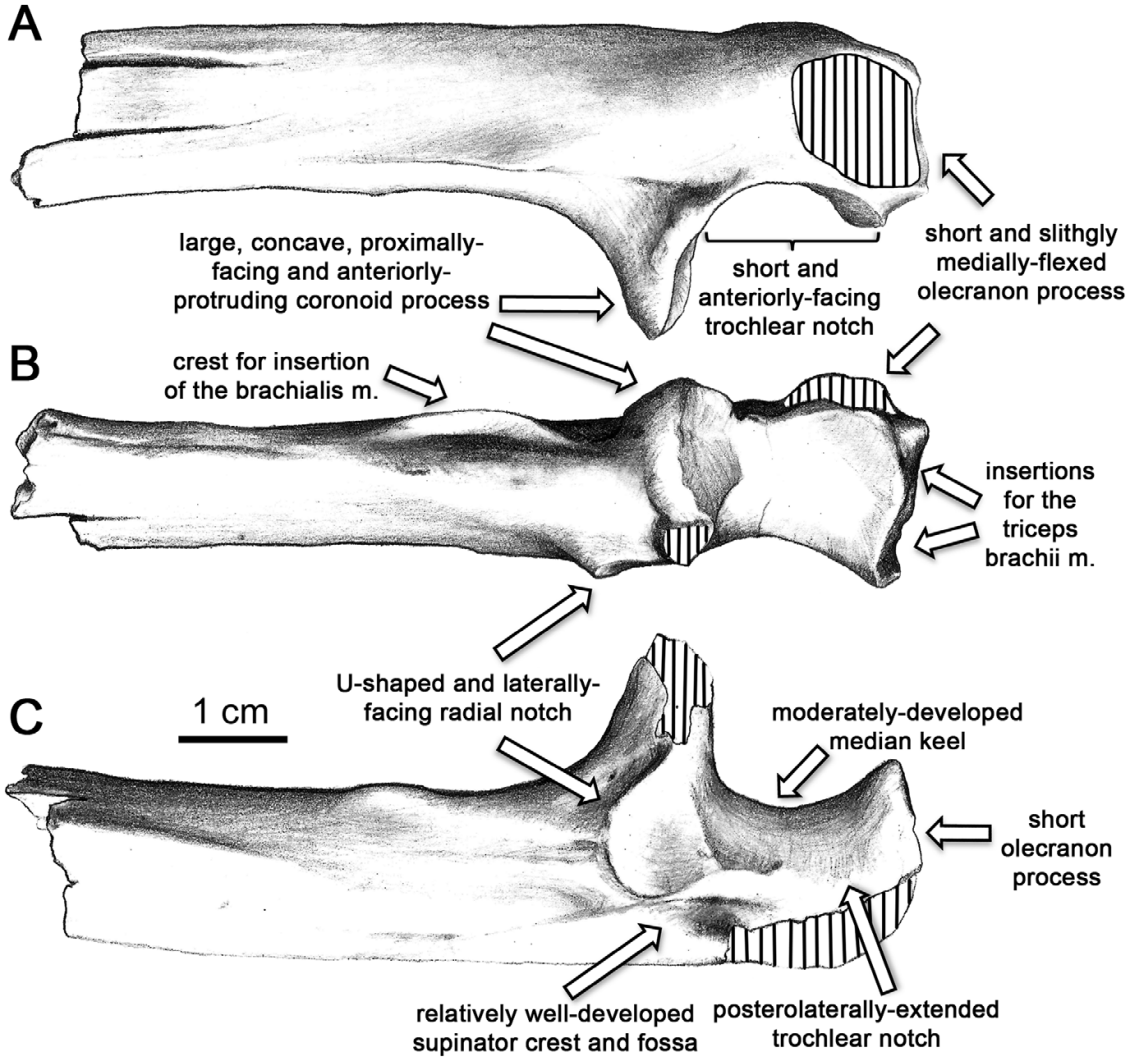
Main morphological features of the *H. laietanus* proximal ulna from CF. A, medial; B, anterior; C, and lateral. Stripes denote damaged areas.

The CF specimen most completely preserves the *Hispanopithecus* proximal morphology of the ulna (Figs. 2A–E, 9), which is very informative for making locomotor inferences. The trochlear notch is short and broader laterally (where it further extends posteriorly onto the shaft), with a moderately-developed median trochlear keel. The coronoid process is large and anteriorly-protruding, with a concave surface facing proximally, like the distolateral portion of the trochlear notch, indicating the presence of a spool-shaped humeral trochlea [45]. The radial notch, situated above a relatively well-developed supinator crest, faces laterally. The quite short olecranon process is somewhat tilted posteromedially. Two distinct ulnar morphotypes can be distinguished amongst Miocene apes (Fig. S2). Proconsulids (*Proconsul*, *Turkanapithecus*; Fig. S2C), equatorins (*Equatorius*, *Nacholapithecus*; Fig. S2E) and the kenyapithecin *Griphopithecus* (Fig. S2D) display a colobine-like, primitive morphology (Fig. S2G), characterized by a narrow trochlear notch with a faint medial keel, a proximally-protruding olecranon, a deep shaft and a downward-sloping coronoid process [38], [42], [46]–[48]. *Turkanapithecus*, *Nacholapithecus* and *Griphopithecus* also display a flat and laterally-facing radial notch, and *Nacholapithecus* further combines an overall primitive morphology with a more anteriorly-directed coronoid process [47], like *Griphopithecus*. Extant hominoids (Figs. S2H–J) differ from the above-mentioned taxa by displaying a more derived morphology, characterized by a wide trochlear notch with a well-developed median keel, a poorly-developed olecranon process, and a large and anteriorly-projecting coronoid process (whose medial portion projects proximally, creating an inverted V-shape).

**Figure 10:**
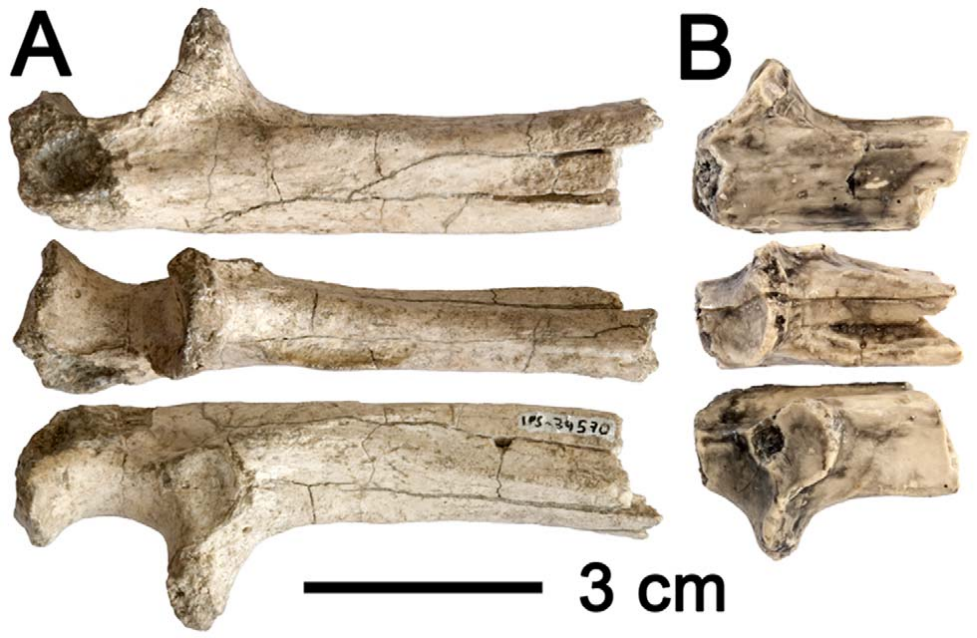
Proximal portion of the ulna of *H. laietanus* and *H. hungaricus*. A, Proximal ulnar fragment of *H. laietanus* IPS34575g from CF1. B, Preserved ulnar portion of *H. hungaricus* RUD 22 from Rudabánya (cast, reversed).

Among Miocene apes, only *Oreopithecus* (Fig. S2C) and to a large extent *Hispanopithecus* (Figs. 3, S2B) display this modern hominoid-like ulnar morphology [42], [45], [49]–[51], whereas *Griphopithecus* (Fig. S2B) displays a more primitive condition (even if incompletely preserved). The CF specimen, however, differs in several respects from *Oreopithecus*, which most closely resembles extant apes by the extremely reduced olecranon process, the short trochlear notch, and the more marked median keel. Overall, the CF specimen most closely resembles the much larger, male proximal ulna of *H. laietanus* from CLL2 [7] and the similarly-sized female partial ulna of *H. hungaricus* from Rudabánya (Fig. 10) [42], [44]. Minor differences with the latter include a more slender proximal shaft and a larger and more anteriorly-protruding coronoid process in the CF specimen, whereas similarities between them include the laterally-facing radial notch, the moderately-developed median keel, and the proximally-facing coronoid process that further defines an inverted V-shape. The two latter features, together with distal humeral morphology, enabled previous authors to infer the presence of a spool-shaped humeral trochlea in *H. hungaricus* [42], [44]. However, unlike the two previously-known specimens, the CF ulna preserves the olecranon process and the proximal portion of the trochlear notch, thus enabling a more complete morphofunctional assessment. Thus, compared to most Miocene apes, *Hispanopithecus* displays a shorter olecranon process together with a shorter and relatively broader trochlear notch. In contrast, the olecranon process of the CF specimen is still somewhat better-developed than in extant apes and *Oreopithecus*, further being somewhat posteromedially flexed–as in previous Miocene apes, extant quadrupedal monkeys and the knuckle-walking African apes, but unlike in hylobatids and orang-utans.

**Figure 11:**
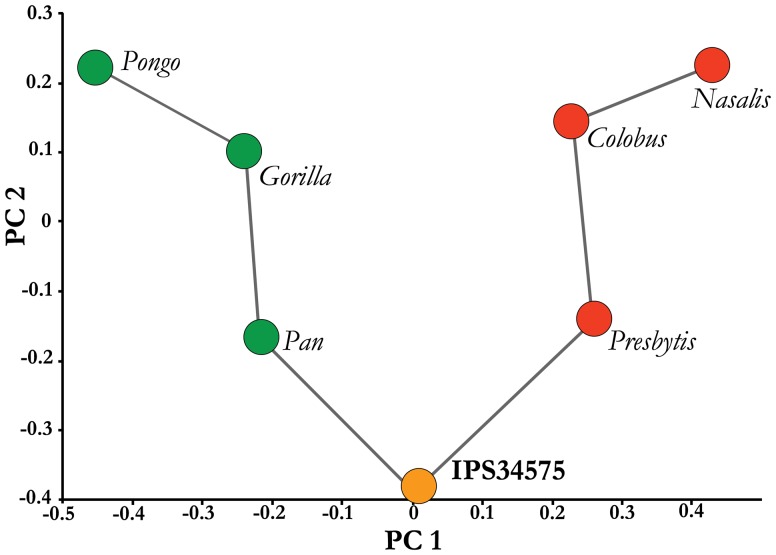
Principal Components Analysis (PCA) of the proximal ulna. This PCA, based on eight shape variables of the proximal ulna (see Materials and methods), shows the phenetic affinities of the CF ulna of *H. laietanus* (in orange) compared to that of selected extant catarrhines (great apes in green, and colobines in red). The two principal components (PC1 and PC2) show that *H. laietanus* displays a proximal ulnar morphology unlike that of extant catarrhines, and somewhat intermediate between that of monkeys and extant apes (see text for further explanation). See PCA results in Table S1.

Finally, a PCA based on eight shape variables of the proximal ulna (Figure 11, Table S1) further confirms that *H. laietanus* displays a proximal ulna unlike that of extant great apes, and intermediate between them and colobines, being most similar to that of *Presbytis* and *Pan*. The PC1, which explains 55.5% of the variance, separates extant great apes from colobine monkeys mainly due to the relatively wider trochlear surfaces and anteroposterior lower proximal shaft of the former, coupled to a lesser degree with the relatively mediolaterally broader proximal shaft and proximodistally shorter radial notches of great apes compared to colobines; along the PC1, the CF proximal ulna falls just in between great apes and colobines. In turn, the PC2, which explains 30.4% of the variance, is basically driven by the anteroposterior diameter of the radial notch, with *Pongo, Gorilla, Nasalis* and *Colobus* displaying relatively anteroposteriorly high radial notches, and IPS34575 falling on the opposite side, by displaying an anteroposteriorly very short radial notch. To a lesser extent, this axis also reflects wider proximal articular breadths (positive values), as well as anteroposteriorly higher proximal shafts, broader proximal articular anteroposterior diameters and deeper sigmoid notches (negative values), with *Pan* and *Presbytis* displaying intermediate values on this axis, although slightly closer to the CF specimen.

## Discussion

### Taxonomic Attribution

Dental comparisons of the CF material with Middle Miocene hominoids from the Vallès-Penedès [9], [21] are restricted to *Anoiapithecus* [35], given the lack of lower teeth for both *Pierolapithecus* [36] and *Dryopithecus* [13]. The CF teeth, however, differ from French *D. fontani* specimens in the same features previously noted to distinguish *Hispanopithecus* species from *Dryopithecus fontani* [10], [14]. Regarding *Anoiapithecus*, it differs from the CF and other *H. laietanus* specimens regarding p4 as well as lower molar morphology and proportions. On the basis of size, proportions and morphology, the CF dental remains fit well into the range of variation of *Hispanopithecus laietanus* [1]–[4], [6], in further agreement with its age (10.0-9.7 Ma) [27], [28], only slightly older than other *H. laietanus* remains (9.7-9.5 Ma), but younger than *H. crusafonti* (10.4-10.0 Ma) [21]. Some authors have favored the distinct species status of *H. crusafonti* [9], [10], [13], [14], [20], [22], [52], at least for the CP material [20], whereas others have considered that both samples are insufficiently distinct [11], [34]. In any case, the CF specimens differ from those of *H. crusafonti* from CP in several respects: the shorter and relatively wider p3, and the narrower buccal cuspulids and more extensive talonid basins of the lower molars. The CF molars further differ from those of TF–tentatively attributed to *H. crusafonti* by some authors [9], [10], [14], [21], but assigned to *Dryopithecus fontani* by others [11], [20], [34]–in the same features. Therefore, the CF remains are best attributed to *H. laietanus*.

### Locomotor Inferences

The partial skeleton from CF provides new information on several anatomical regions, such as the first rib, the acromial end of the clavicle and the proximal ulna, which were previously unknown in the partial skeleton from CLL2 [7], thus enabling us to refine previous locomotor inferences for this taxon. The new remains agree well with previous inferences of an orthograde body plan in this taxon [7], as shown among others by the various modern hominoid-like features displayed by the first rib fragment, which represents the first direct evidence of thorax morphology in *Hispanopithecus*. However, both the rib and the clavicular fragments display a mixture of primitive (monkey-like) and derived (modern hominoid-like) features, suggesting that *H. laietanus* possessed a locomotor repertoire unlike that of extant hominoids. In this regard, the proximal morphology of the ulna recorded by the CF skeleton is most significant, given the fact that modern hominoids are characterized by a distinctive elbow morphology.

The proximal ulnar morphology shared by extant hominoids is functionally related to increased pronation/supination and flexion/extension ranges, by providing substantial stability without compromising mobility at the humeroantebranchial joint [42], [51], [53]–[59]. In contrast, the ulna of Early and Middle Miocene apes resembles extant non-hominoid anthropoids, reflecting a more restricted range of flexion/extension, and a greater stability only in full pronation [55]. In contrast, the universal stability attained by the elbow of extant apes under a broad range of positions is suitable for extensive forelimb use under both tension and compression during eclectic climbing and below-branch suspensory behaviors [42], [56]. The narrow and anteroposteriorly deep proximal ulnar shaft of Early and Middle Miocene hominoids, together with their longer olecranon process–where the principal elbow extensor inserts [60]–and downward-sloping coronoid process, suggest stronger bending stresses along the parasagittal plane with a primarily semiflexed elbow (i.e., a limited range of extension), and are therefore indicative of quadrupedalism [42], [46], [61]–[63]. Nevertheless, proconsulids, afropithecids and kenyapithecines already display a mosaic of primitive anthropoids and some derived hominoid features [43], [62], [64], indicating that the elbow joint was loaded in a variety of flexion/extension and pronation/supination postures, even though higher stability was still attained in full pronation [43], [55], [65]. In the ulna, the higher degree of forearm rotation of Miocene apes is reflected in their more laterally facing radial notch–an anteriorly-facing radial notch being related to habitually pronated forearms [42], [55], [63], [65]–as well as in their stronger muscular insertions–related to enhanced supination capabilities [45]. Together with other anatomical regions, the elbow of these taxa suggests that they were slow-moving, above-branch pronograde quadrupeds with no suspensory adaptations, but already employing more abducted limb postures and more powerful grasping capabilities than other anthropoids [25], [43], [55], [62]–[66]. Amongst Middle Miocene African hominoids, *Nacholapithecus* most clearly shows a humeroulnar complex somewhat more derived towards a higher stability against mediolateral stresses and a somewhat enhanced pronation/supination range, probably indicating a higher reliance on climbing than in previous taxa, in spite of still lacking suspensory adaptations [40], [43], [47], [66], [67]. A similar condition is displayed by the proximal ulna of *Griphopithecus* [10], [42], [43], [68], as shown by the still narrow trochlear notch with no median keel and the long olecranon process.

The ulna is unknown for the stem pongine *Sivapithecus* and the putative stem hominids *Pierolapithecus* and *Dryopithecus*, but other postcranial evidence suggests that these taxa displayed unique locomotor repertoires, currently unknown amongst extant apes, combining powerful-grasping, pronograde quadrupedalism with some orthograde behaviors but with no suspensory adaptations [25], [26], [36], [43], [62], [69]–[72]. Amongst Miocene apes, only the Late Miocene *Oreopithecus* displays a fully modern-hominoid-like elbow joint, as shown by the very short olecranon process and marked trochlear keel [43], [49]–[51], [54], [66], [69], [73]. *Hispanopithecus*, however, first documents undoubted adaptations to below-branch suspensory behaviors, including relatively long forelimbs [7], long and curved phalanges [7], [8], [25], [74], femoral morphology [7], [23] and femoral neck cortical thickness distribution [25]. *Hispanopithecus* is therefore a key taxon for understanding the emergence of modern locomotor behaviors amongst hominoids. The modern elbow morphology of *H. hungaricus* from Rudabánya had been interpreted as suitable for preserving joint stability in all positions along the full broad range of flexion/extension, and enabling a broad range of pronation/supination [12], [42]–[44]. This is most clearly shown by the orientation and development of the coronoid process (indicative of a spool-shaped humeral trochlea) and the relatively reduced olecranon process of the CF ulna, which provide stability during rotatory movements and further allow for full extension of the elbow during suspensory behaviors [45], [51], [54], [55]. Hence, the CF specimen agrees with previous assessments based on the spool-shaped trochlea of *H. hungaricus* [10], [42], [44], and further reinforces previous inferences of suspensory behaviors in *H. laietanus* [7], [8], [24], [25], [74].

At the same time, the CF specimen also shows that *Hispanopithecus* still retained a proximal ulnar morphology unlike that of extant hominoids, suggesting the presence of significant differences in their locomotor repertoires. On the one hand, the PCA reported in this paper indicates that the CF proximal ulna is morphologically distinctive, and intermediate between that of great apes and colobine monkeys in several regards (Figure 11). Thus, the distinctive anteroposteriorly short radial notch of the CF specimen (as shown by the PC2), coupled with its intermediate proximodistal length (as depicted in the PC1), are reflecting the U-shaped articular surface characteristic of most Miocene apes. The CF specimen is also intermediate regarding anteroposterior shaft and articular diameters at the proximal ulna, with monkeys displaying the highest diameters. This has been related to higher bending stresses on this plane, in relation to predominant parasagittal limb movements [42], and might also be linked to the relatively slender ulnae in comparison to the radius of monkeys compared to apes, further reflecting the higher mediolateral bending stresses of the former, in relation to a predominant quadrupedal posture [75]. *Hispanopithecus* further retained a somewhat proximally-projecting and posteromedially-tilted olecranon process. Olecranon orientation relative to the forearm determines the elbow position at which the maximum mechanical advantage of the triceps brachii muscle is attained [60]. Therefore, the slightly proximally-protruding olecranon process of *Hispanopithecus* may be functionally explained by the retention of pronograde behaviors, which require elbow stability also at semiflexed postures [60]. It should be taken into account that the *Hispanopithecus* olecranon process is also medially protruding, thus more closely resembling the condition displayed by African apes among extant hominoids [76], [77]. This condition, termed ‘flexor expansion’ [77], has been related to the role played by the digital flexors during knuckle-walking [77]. Although such functional relationship remains to be tested, the absence of this feature in orangutans [76] and the presence in monkeys and Miocene apes suggests that it might be related to quadrupedal postures in general. Whereas knuckle-walking adaptations can be discounted in *H. laietanus*, the proximomedial expansion of its ulna is suggestive of a higher degree of quadrupedalism than in hylobatids and *Pongo*, and thefore agrees with the presence of palmigrady-related features in the hand of this taxon–the short metacarpals and the morphology of the proximal articulation of the proximal phalanges [8]–although to a lesser extent than in *Pierolapithecus* and other Middle Miocene taxa [25], [26]. Powerful grasping capabilities, suitable for above-branch quadrupedalism, can be also inferred for *H. hungaricus* on the basis of carpal and phalangeal morphology, suggesting the presence of a large and powerful pollex– as in other Miocene apes [18], [72], [78]. A significant amount of quadrupedalism is further indicated by the peculiar (Miocene ape-like) configuration of the shoulder girdle and the mediolaterally-compressed shaft of the radius from the CF skeleton. In summary, new evidence provided here confirms that the Late Miocene great ape *Hispanopithecus* displayed an adaptive compromise between hyperextension capabilities (presumably for suspensory and other orthograde behaviors) and more primitive, pronograde behaviors.

### Implications for the Evolution of Crown-hominoid Positional Behaviors

Despite phylogenetic uncertainties, *Hispanopithecus* is considered a crown-hominid by most researchers, being alternatively interpreted as a stem pongine [5], [6] (an extinct taxon more closely related to orangutans than to African apes and humans), a stem hominine [10], [12], [22] (more closely related to the African ape and human clade than to orangutans), or a stem hominid [9], [21], [35] (a fossil great ape preceding the divergence between pongines and hominines, but postdating the split between hylobatids and the great ape and human clade)–see ref. [9] for further discussion on hominoid systematics and the arguments put forward in favor of each of these phylogenetic alternatives for *Hispanopithecus*. From a locomotor viewpoint, *Hispanopithecus* is the oldest ape documenting unquestioned suspensory adaptations, shared by all extant crown hominoids (hylobatids and hominids), thus being of utmost significance for understanding the emergence of modern hominoid positional behaviors. The proximal ulna from CF, being the most complete available for the genus *Hispanopithecus*, reflects an elbow complex suitable for preserving stability along the full range of flexion/extension and enabling a broad range of pronation/supination, thus confirming previous inferences of specialized suspensory behaviors [7], [8], [23]–[25], [74]. However, the rib, clavicular and scapular remains display a mixture of primitive and derived features, suggesting that *Hispanopithecus*, in spite of orthograde features, possessed a locomotor repertoire currently unknown among extant hominoids. This is further confirmed by the CF ulna, which differs from that of the committed suspensory hylobatids and orang-utans in the slightly more proximally projected olecranon. The latter is functionally interpreted as a compromise between enhanced extension at this joint for suspensory behaviors and for still important weight-bearing postures with a semi-flexed elbow during above-branch arboreal quadrupedalism. Thus, during quadrupedalism *Hispanopithecus* would not have displayed the fully-extended elbow position most commonly employed by extant hominoids. African apes display a similar morphology (medially but not proximally protruding olecranon) due to adaptation to knuckle-walking, which represents a compromise between terrestrial quadrupedal behaviors–with extended elbow postures [63]–and orthograde arboreal behaviors. However, knuckle-walking can be discounted in *Hispanopithecus* on the basis of phalangeal and metacarpal morphology [8], [18], [25]. The CF proximal ulna therefore reinforces the view [8], previously dismissed by other authors [19], that the *Hispanopithecus* forelimb reflects a different locomotor compromise, combining climbing and suspensory behaviors with powerful-grasping above-branch palmigrady.

The possession in fossil apes of locomotor repertoires unknown among extant taxa agrees well with the inferred mosaic evolution of the hominoid locomotor apparatus [8], [25], [26], [43], [62], [64], [66], [73], but has profound implications for the reconstruction of ancestral locomotor repertoires. The lack of suspensory adaptations in the orthograde, putative stem hominid *Pierolapithecus* [25], [26], [36], [66], [71]–see [74] for a different interpretation–otherwise adapted to vertical climbing and powerful-grasping palmigrady, suggests that suspensory behaviors evolved independently at least between hylobatids and hominids [9], [25], [26], [36], [66]. Such a contention is reinforced by lack of suspensory adaptations in the pongine *Sivapithecus*, despite possessing a modern elbow configuration with a spool-shaped trochlea [43], [70]. *Hispanopithecus*, however, stands out as the only Miocene ape in which palmigrady-related features are retained *in spite* of clear-cut suspensory adaptations. Such a locomotor mosaic is unknown not only among extant, but also among other fossil apes. Given that suspensory features have independently evolved in other primates [43], [64], [65], [68], [73], most notably atelines [79], their independent evolution in several crown hominoid lineages, from an orthograde ancestor similar to *Pierolapithecus*, does not seem unlikely. Atelines display a combination of climbing, quadrupedal and suspensory behaviors, but lack several modern-hominoid postcranial adaptations, such as the characteristic hominoid humeroantebrachial complex that provides universal stability at the elbow joint under a variety of positions [43], [56]. These features, such as the spool-shaped humeral trochlea, are useful during suspensory behaviors for resisting the mediolateral stresses caused by strong wrist and finger flexor muscles [62]. Nevertheless, they could have originally evolved for stabilizing the humerulnar joint during above-branch quadrupedalism [43], [65], i.e. as an adaptation to increase pronation-supination forearm capabilities for maintaining balance above arboreal supports, as required by the tailless hominoid condition [62]–[63], [66], [80].

*Hispanopithecus* differs from other Miocene apes by uniquely showing a transitional stage in which a modern hominoid-like elbow complex appears to be simultaneously an adaptation to keep balance during palmigrady as well as an exaptation for performing suspensory behaviors. The latter eventually replaced above-branch quadrupedalism in all extant ape lineages, ultimately enabling great apes to reach very large body masses that would have been otherwise untenable. Nevertheless, given its quite large body size, the retention of above-branch quadrupedalism in *Hispanopithecus* suggests that suspensory behaviors did not originally evolve to solve balance problems during horizontal arboreal travel. More specific targets of selection, such as a more efficient feeding on terminal branches in spite of large body size [8], [66], could have been involved. If so, the modern-hominoid elbow morphology could have been co-opted several times independently from a partly quadrupedal ancestor–at least hylobatids and hominids, but perhaps even hominines, pongines and/or dryopithecines–in order to perform these behaviors [25], [43], [66]. At the very least, the unique locomotor repertoire evidenced by *Hispanopithecus* should warn us against reconstructing the ancestral positional behaviors of extant hominoid subclades on the basis of the biased evidence provided by their few and very specialized remaining living representatives, without taking the fossil evidence into account.

## Materials and Methods

### Body Mass Estimation

Body mass (BM, in kg) was estimated on the basis of ulnar articular measurements and radial diaphyseal measurements [81] using allometric techniques [31]. Ulnar trochlear surface area (UTSA, in mm^2^) was used as a BM estimator, being computed according to the following equation [31]: UTSA  =  UTSI* x UTML* x acos (1-((2 x UTDP*)/UTSI*)), where UTML* (in mm) is the proximal ulnar articular surface (trochlear notch) mediolateral dimension, UTSI* (in mm) is the proximal ulnar articular surface (trochlear notch) superoinferior dimension, and UTDP* (in mm) is the proximal articular ulnar articular surface (trochlear notch) depth. Furthermore, radial midshaft average diameter (R50AB, in mm) was also employed as a BM estimator, being computed as the average between the anteroposterior (R50AP) and mediolateral (R50ML) diameters [31].

### Morphometric Analysis of the Proximal Ulna

In order to quantify the phenetic affinities of the proximal ulna, we relayed on the published means of the following eight measurements from this anatomical region in extant great apes and selected colobines (the most arboreal catarrhines), extracted from Table 4C in ref. [42]: PAP, proximal shaft height (anteroposterior); PSML, proximal shaft mediolateral diameter; PAB, proximal articular breadth; TAB, trochlear articular breadth; RAP; radial notch anteroposterior diameter; RPD, radial notch proximodistal diameter; PAAD, proximal articular anteroposterior diameter; SND, sigmoid notch depth. Based on these linear measurements, we created eight Mosimann shape variables by dividing each raw measurement by the geometric mean of all the original variables and applying a logarithmic transformation (with natural logarithms, ln) [82], [83]. We summarize these log-shape data via Principal Components Analysis (PCA) of the covariance matrix and a minimum-spanning tree based on Euclidean distances, using the software Palaeontological Statistics (PAST) [84].

## Supporting Information

Figure S1 **Morphology of the distal humeral diaphysis of** ***H. laietanus*** **compared to selected hominoids.** Each specimen depicted (from left to right) in anterior, medial, posterior and lateral views. A, *H. laietanus* female IPS34575i; B, cf. *Dryopithecus fontani* IPS4334 male (reversed); C, *D. fontani* HGP 3 female (cast); D, *Griphopithecus darwini* 1991/580 (cast, reversed); E, *Proconsul heseloni* KNM RU 2036 AH (cast); F, *Sivapithecus indicus* GSP 30730; G, *Hylobates syndactylus* AMNH 106581 (reversed); H, *Pongo pygmaeus* female; I, *P. pygmaeus* male.
(TIF)

Figure S2 **Morphology of the proximal ulnar morphology of** ***H. laietanus*** **compared to selected hominoids.** Each specimen depicted (from top to bottom) in medial, anterior and lateral views. All specimens depicted as left and not to scale (scale bars correspond to 3 cm). A, *H. laietanus* IPS34575g; B, *H. hungaricus* RUD 22 (cast, reversed); C, *Oreopithecus bambolii* IGF 11778 (cast, reversed); D, *Griphopithecus darwini* 1992/581 (cast); E, *Nacholapithecus kerioi* KNM-BG 32250; G, *Proconsul nyanzae* KNM RU 1786 (cast); G, *Nasalis larvatus* AMNH106272; H, *Hylobates syndactylus* AMNH106581; I, *Pongo pygmaeus* AMNH200900CA; J, *Pan troglodytes* AMNH174860. Photographs depicted in (E) were kindly provided by Masato Nakatsukasa.
(TIF)

Table S1 **Results of the Principal Components Analysis (PCA) of the proximal ulna.** This PCA analysis is based on eight Mosimann shape variables, computed from the mean values for the following eight linear measurements [42], by dividing them by their geometric mean (GM) and applying logarithms (ln): PAP, proximal shaft height (anteroposterior); PSML, proximal shaft mediolateral diameter; PAB, proximal articular breadth; TAB, trochlear articular breadth; RAP; radial notch anteroposterior diameter; RPD, radial notch proximodistal diameter; PAAD, proximal articular anteroposterior diameter; SND, sigmoid notch depth. Only those PCs explaining more than 1% of variance have been depicted. The first (PC1) and second (PC2) principal components (see Figure 11) explain more than 85% of the variance. See main text for a morphofunctional interpretation.
(DOCX)

Text S1 **Description of dentognathic and postcranial remains of** ***Hispanopithecus laietanus*** **from CF.**
(PDF)
