# Supplementary figures and images for: A Partial Skeleton of the Fossil Great Ape Hispanopithecus laietanus from Can Feu and the Mosaic Evolution of Crown-Hominoid Positional Behaviors
Source: PLoS One. 2012 Jun 25;7(6):e39617. doi: 10.1371/journal.pone.0039617 (PMC3382465; doi:10.1371/journal.pone.0039617)

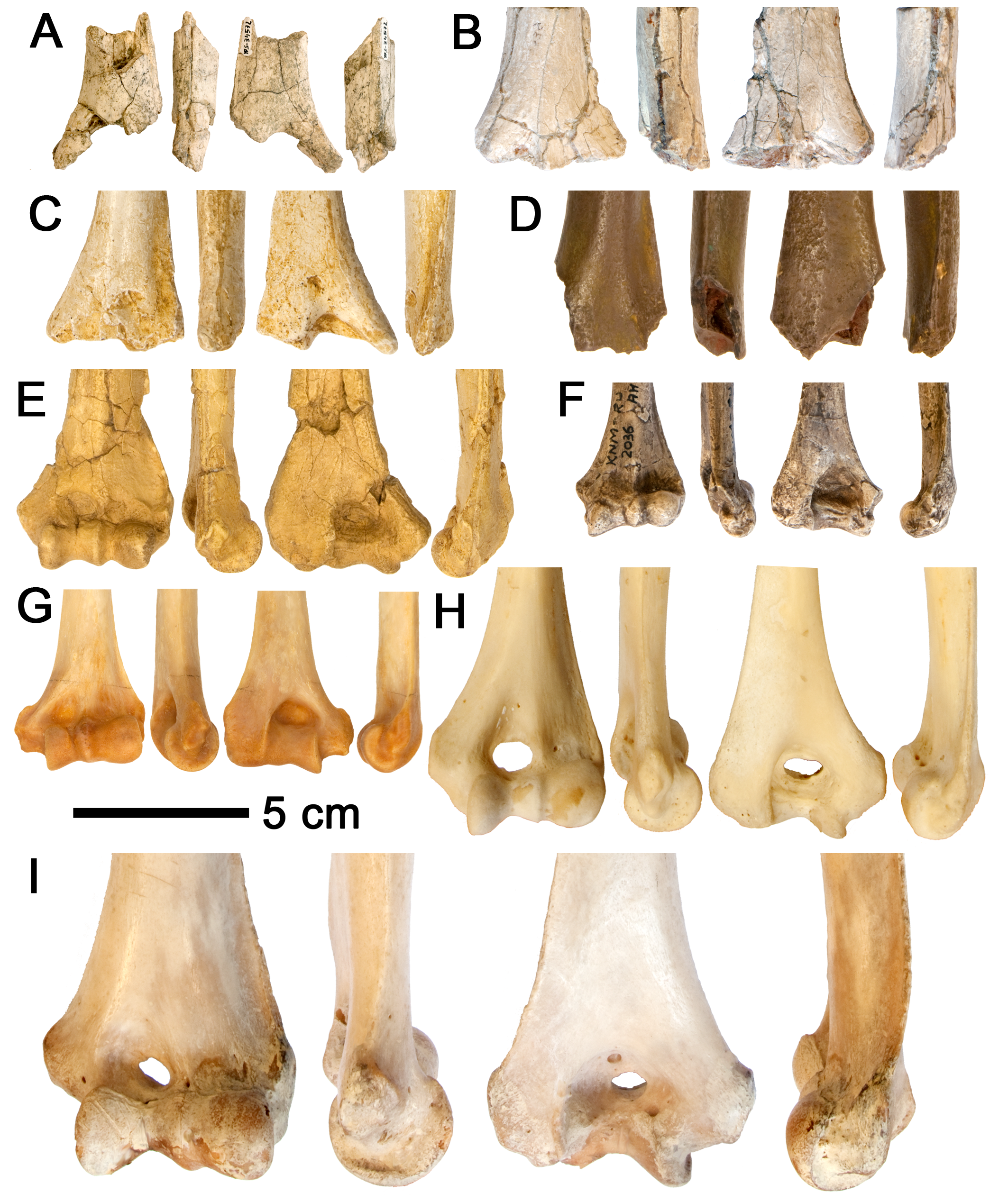

Supplement: Figure S1 — Morphology of the distal humeral diaphysis of H. laietanus compared to selected hominoids. Each specimen depicted (from left to right) in anterior, medial, posterior and lateral views. A, H. laietanus female IPS34575i; B, cf. Dryopithecus fontani IPS4334 male (reversed); C, D. fontani HGP 3 female (cast); D, Griphopithecus darwini 1991/580 (cast, reversed); E, Proconsul heseloni KNM RU 2036 AH (cast); F, Sivapithecus indicus GSP 30730; G, Hylobates syndactylus AMNH 106581 (reversed); H, Pongo pygmaeus female; I, P. pygmaeus male. (TIF) [file pone.0039617.s001.tif]

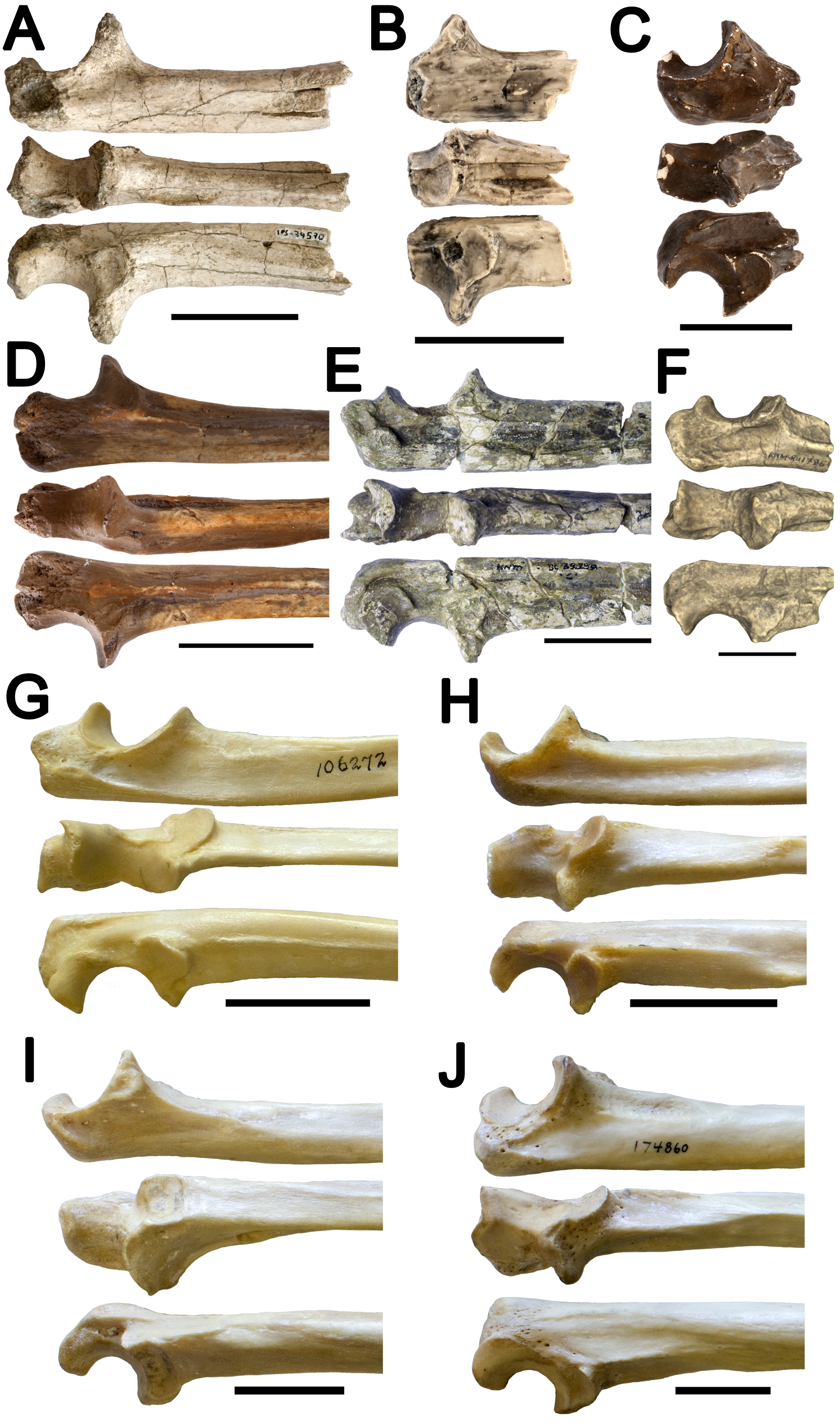

Supplement: Figure S2 — Morphology of the proximal ulnar morphology of H. laietanus compared to selected hominoids. Each specimen depicted (from top to bottom) in medial, anterior and lateral views. All specimens depicted as left and not to scale (scale bars correspond to 3 cm). A, H. laietanus IPS34575g; B, H. hungaricus RUD 22 (cast, reversed); C, Oreopithecus bambolii IGF 11778 (cast, reversed); D, Griphopithecus darwini 1992/581 (cast); E, Nacholapithecus kerioi KNM-BG 32250; G, Proconsul nyanzae KNM RU 1786 (cast); G, Nasalis larvatus AMNH106272; H, Hylobates syndactylus AMNH106581; I, Pongo pygmaeus AMNH200900CA; J, Pan troglodytes AMNH174860. Photographs depicted in (E) were kindly provided by Masato Nakatsukasa. (TIF) [file pone.0039617.s002.tif]
