# Supplementary material for: A Partial Skeleton of the Fossil Great Ape Hispanopithecus laietanus from Can Feu and the Mosaic Evolution of Crown-Hominoid Positional Behaviors
Source: PLoS One. 2012 Jun 25;7(6):e39617. doi: 10.1371/journal.pone.0039617 (PMC3382465; doi:10.1371/journal.pone.0039617)
Supplement: Table S1 — Results of the Principal Components Analysis (PCA) of the proximal ulna. This PCA analysis is based on eight Mosimann shape variables, computed from the mean values for the following eight linear measurements [42], by dividing them by their geometric mean (GM) and applying logarithms (ln): PAP, proximal shaft height (anteroposterior); PSML, proximal shaft mediolateral diameter; PAB, proximal articular breadth; TAB, trochlear articular breadth; RAP; radial notch anteroposterior diameter; RPD, radial notch proximodistal diameter; PAAD, proximal articular anteroposterior diameter; SND, sigmoid notch depth. Only those PCs explaining more than 1% of variance have been depicted. The first (PC1) and second (PC2) principal components (see Figure 11) explain more than 85% of the variance. See main text for a morphofunctional interpretation. (DOCX) [file pone.0039617.s003.docx]

**Table S1 Results of the Principal Components Analysis (PCA) of the proximal ulna.** This PCA analysis is based on eight Mosimann shape variables, computed from the mean values for the following eight linear measurements [42], by dividing them by their geometric mean (GM) and applying logarithms (ln): PAP, proximal shaft height (anteroposterior); PSML, proximal shaft mediolateral diameter; PAB, proximal articular breadth; TAB, trochlear articular breadth; RAP; radial notch anteroposterior diameter; RPD, radial notch proximodistal diameter; PAAD, proximal articular anteroposterior diameter; SND, sigmoid notch depth. Only those PCs explaining more than 1% of variance have been depicted. The first (PC1) and second (PC2) principal components (see Figure 11) explain more than 85% of the variance. See main text for a morphofunctional interpretation.

|  | **PC1** | **PC2** | **PC3** | **PC4** | **PC5** |
| --- | --- | --- | --- | --- | --- |
| **Eigenvalue** | 0.10164 | 0.05555 | 0.01472 | 0.00878 | 0.00188 |
| **% variance** | 55.557 | 30.364 | 8.045 | 4.802 | 1.027 |
|  | **PCA loadings** | | | | |
| **Shape variable** | **PC1** | **PC2** | **PC3** | **PC4** | **PC5** |
| ln (PAP/GM) | 0.4674 | -0.3500 | 0.3529 | -0.0479 | 0.0361 |
| ln (PSML/GM) | -0.3232 | 0.0158 | -0.2142 | -0.8432 | 0.0471 |
| ln (PAB/GM) | -0.2660 | 0.3480 | 0.2823 | 0.1956 | 0.4765 |
| ln (TAB/GM) | -0.6106 | -0.0981 | -0.1331 | 0.4490 | -0.2572 |
| ln (PAAP/GM) | 0.0401 | -0.3243 | 0.0720 | 0.0099 | -0.6095 |
| ln (SND/GM) | 0.0164 | -0.3250 | 0.2620 | 0.0407 | 0.4132 |
| ln (RAP/GM) | 0.2993 | 0.7320 | 0.1713 | -0.0157 | -0.3318 |
| ln (RPD/GM) | 0.3767 | 0.0017 | -0.7931 | 0.2116 | 0.2256 |
