# Supplementary material for: A Partial Skeleton of the Fossil Great Ape Hispanopithecus laietanus from Can Feu and the Mosaic Evolution of Crown-Hominoid Positional Behaviors
Source: PLoS One. 2012 Jun 25;7(6):e39617. doi: 10.1371/journal.pone.0039617 (PMC3382465; doi:10.1371/journal.pone.0039617)
Supplement: Text S1 — Description of dentognathic and postcranial remains of Hispanopithecus laietanus from CF. (PDF) [file pone.0039617.s004.pdf]

Text S1 Description of dentognathic and postcranial remains of *Hispanopithecus laietanus* from CF.

Supporting Information to: A Partial Skeleton of the Fossil Great Ape *Hispanopithecus laietanus* from Can Feu and the Mosaic Evolution of Crown-Hominoid Positional Behaviors.

David M. Alba, Sergio Almécija, Isaac Casanovas-Vilar, Josep M. Méndez and Salvador Moyà-Solà

Description of dentognathic remains

The mandible is preserved broken into several tooth-bearing fragments, various isolated teeth and a fragment of mandibular ramus and associated condyle (IPS34575n) (see Table 2 for further details). The bony portions are too incomplete to provide relevant information, so that only dental morphology is described below (Fig. 1).

Lower central incisor

The right i1 IPS34575c (Figs. 1A-D, 5A) preserves the crown with some dentine exposure at the apical edge and almost the complete root. It is a spatulate and waisted tooth, the crown being slightly more expanded distally than mesially, with a moderately convex labial profile and a concave lingual contour. There is a poorly-defined lingual pillar and faint mesial and distal ridges, which converge to a bulbous but diffuse basal bulge. The root is straight and mesiodistally compressed, being inflated towards its basal portion, and progressively tapering apically. The cementoenamel junction displays a V-shape that is more marked distally than mesially.

Lower third premolars

Both the right (IPS34575e) and left (IPS34575d) p3 are available (Figs. 1E-N, 6A-B), the left one more completely preserving the mesial root, which is partly fused with the distal one on its basalmost portion. Both specimens show some wear at the single main cuspid (with minimal dentine exposure on the right specimen) and the mesial and distal portions of the crown. It is a sectorial tooth with a suboval occlusal outline (much longer than broad), with a wide mesiobuccal wear facet against the upper canine,

indicating the existence of a well-developed canine honing facet. The convex buccal crown portion is separated from the more concave portion by two cristids of mesiolingual and distobuccal direction that run from the main cuspid. A restricted distal fovea is separated from the lingual crown portion by a third, irregular cristid of distal direction, which runs towards the distal marginal ridge without merging with it. The distal margin is continuous with the moderately-developed lingual cingulid than runs until the mesiolingual corner of the crown.

Lower fourth premolars

The left p4 IPS34575b (Figs. 1O-Q, 7A), still partially socketed into a fragment of mandibular bone, preserves the crown with a moderate degree of wear (no dentine exposure) and most of the roots (except for the tips). The distal root is more slender than the mesial one, to which it is fused on their basalmost portion. The crown displays a suboval outline, being slightly longer than broad, and broader mesially than distally. It displays two mesial main cuspids, situated quite close to one another and united by a short transverse cristid. The latter separates the subquadrangular mesial fovea, mesially closed by a marked mesial marginal ridge, and situated towards the lingual portion of the crown, from the much deeper and more extensive talonid basin. There are two secondary cuspulids situated at the two distal corners of the crown: the entoconid, situated quite close to the metaconid, and the hypoconid (eroded by wear), situated farther from the protoconid.

Lower molars

Both the right (IPS34575a) and left (IPS34575f) molar rows are preserved (Figs. 1R-W, 8A-B). These teeth show a quite advanced degree of wear, progressively decreasing from m1 to m3: thus, there is dentine exposure at the apices of the three buccal cuspids in the m2 and m3, and at the tips of the five main cuspids of the m1 (where dentine exposure is continuous along the three buccal cuspids). The three molars display a subrectangular occlusal outline somewhat constricted between the mesial and distal lobes, thus resulting in a slightly biconvex buccal and lingual profiles. The m1 is smaller and relatively narrower than the m2, whereas the m3 resembles in length the m2 but is also relatively narrower. Due to wear, not much of occlusal morphology can be

ascertained. The lingual cuspids are more peripheralized than the buccal ones. The protoconid is slightly more mesially situated than the metaconid, which is apparently the most protruding cuspid, and the hypoconid is clearly more mesially situated than the entoconid. The hypoconulid is clearly situated on the buccal moiety of the crown, although closer to crown midline than the remaining buccal cuspids. At least on the m3, there is a secondary cuspulid just mesial to the entoconid at the end of the postmetacristid. Remnants of the transverse grooves separating the bases of the main cuspids remain, the metaconid base contacting with that of the hypoconid, thus displaying a Y5 occlusal pattern. The mesial fovea must have been very short, thus contrasting with the much more extensive and deeper talonid basin, whereas the distal fovea is very restricted and situated on the lingual portion of the crown; the latter might have been separated from the talonid basin by a transverse groove connecting the hypoconulid with the entoconid, although this cannot be definitively ascertained due to wear. No cingulids are present. On the left molar row, it can be observed that the mesial roots of the m1 are fused into a single one, just like the mesial roots of the m3 and the distal roots of the m2; the distal lobe of the m3 displays a single, stouter root that is twisted distally.

Description of postcranial remains

Scapula

Only two small fragments of left scapula (IPS34575m) are available, including a fragment of scapular blade (Figs. 2B'), as well as a lateral fragment of acromion process (Figs. 2C'-F'). The scapular blade fragment has a length of 35.5 mm and a width of 14.9 mm. It preserves the medial portion of the scapular spine until the medial border of the scapula (only minimally eroded). The spine is straighter than in extant hominoids and less obliquely directed towards the medial border, suggesting a more monkey-like shape of the scapula as a whole, although this is difficult to evaluate given the small portion that is preserved. The fragment of acromial process, which measures 21.5 mm in length, is more compressed than in monkeys (10.5 x 6.8 mm at the midpoint of the preserved fragment), apparently resembling in shape, although being somewhat shorter than, that of extant hominoids.

Clavicle

Only the most lateral (acromial) portion of the left clavicle (IPS34575l) is preserved (Figs. 2R-U), with a length of 39.2 mm and a maximum anteroposterior width of 12.3 mm. The acromial end is superoinferiorly narrow (relative to the shaft) and slightly tilted posteriorly, whereas the shaft outline visible due to breakage displays an ellipsoidal diameter (greater in craniocaudal direction). The cranial surface is flat; it displays a marked ridge on the posterior side for the attachment of the trapezius muscle, and a marked rugosity on its anterior border for the attachment of the deltoideus muscle. On the posteroinferior side, the conoid tubercle is very conspicuous and quite laterally situated, and the deltoid tubercle, on the anterior side, is wide, both delimiting a wide groove just anterior to it, which may be interpreted as the subclavian groove. Continuing the conoid tubercle on the posterior side, there is the oblique line for the trapezoid line, which is very soon truncated by the acromial end. Overall, the preserved portion is quite straight, thus differing from the condition of extant great apes, which display a marked sigmoid curvature. The preserved portion is very slender and straight, being more monkey-like than ape-like, although it is most similar to the partial clavicle of *Equatorius* [1,2].

First rib

Only the most proximal portion of the right first rib (IPS34575k) is preserved (Figs. 2M-Q), with a maximum anteroposterior width of 14.4 mm and a craniocaudal height of 4.9 mm, including the tubercle, the beginning of the neck and the proximal shaft. The tubercle is protuberant, like in monkeys, hylobatids and humans, and displays an articular facet on its most proximal end, which is oriented slightly cranially and displays and oval outline. The neck is also slightly tilted cranially, but aligned with the shaft to a large extent. The remaining portion of the shaft is uniformly compressed craniocaudally. The relative position of the tubercle and neck, and the angle they define, resembles that in hylobatids, African apes and humans, while the angle between the neck and the shaft is higher in monkeys and *Pongo*.

Humerus

This bone is only very partially preserved, including several cortical fragments of the diaphysis (IPS34575i) as well as the distalmost portion of the left shaft (IPS34575i; Figs. 2V-A’, S1A). The latter is broken at the level of the proximal portion of the olecranon fossa, so that the whole trochlear region, the epicondyles and the radial and coronoid fossae are missing. The preserved portion of the shaft displays a subtriangular transverse outline, being markedly convex anteriorly and more flattened posteriorly. Towards distal, the shaft becomes anteroposteriorly compressed (with its posterior portion being flat or even slightly concave) as well as mediolaterally expanded (resulting in a concave medial profile), being about twice broader than high. The posterolateral portion of the shaft next to the olecranon fossa is very wide and relatively flat, being flanked by a sharp lateral supracondylar crest. In contrast, the posterior shaft portion medial to the olecranon fossa is much narrower and more rounded, and there is no discernible medial supracondylar crest.

Radius

The diaphysis of the right radius (IPS34575h) is preserved (Figs. 2G-J) in two fragments (a longer proximal one, and a shorter distal one), with a total length of 96.7 mm. The shaft is mediolaterally compressed, displaying a subtriangular, almost pyriform outline (rounded posteriorly and ridge-like anteriorly) on the proximal end (anteroposterior height 13.2 mm, mediolateral breadth 8.5 mm), and an elliptical profile on the distal end (anteroposterior height 12.2 mm, mediolateral breadth 9.3 mm). Midshaft diameters of IPS34575h can be estimated at 11.4 mm (anteroposterior) and 9.2 mm (mediolateral). This specimen preserves approximately the same portion of radial diaphysis than the fragment from the *H. laietanus* male partial skeleton IPS18800, which is longer (116.6 mm) and displays larger estimated midshaft diameters (15.1 mm anteroposterior, and 11.2 mediolateral). Both specimens differ from extant hominoids, and resemble quadrupedal monkeys, by displaying a mediolaterally-compressed shaft (instead of the rounder profile displayed by living apes).

Ulna

The ulna is preserved in two fragments: the proximal portion of the left ulna (IPS34575g), including the articular region and proximal shaft (Figs. 2A-F, 9, S2A);

and a distal fragment of a shaft (IPS34575j; Figs. 2K-L). The latter is a short fragment (44.8 mm in length) that tapers distally: the proximal portion shows a slightly elliptical profile (10.5 mm anteroposteriorly, 9.1 mm mediolaterally), with an anterior crest identified as the insertion for the pronator quadratus [3]; in contrast, the distal contour is rounder (8.3 mm anteroposteriorly, 8.0 mm mediolaterally), corresponding to the portion of the shaft just proximal to the distal ulnar articulation. The proximal portion (preserved length 82.7 mm) is most informative, completely preserving the proximal ulnar morphology amongst available *Hispanopithecus* remains. There is some abrasion in both the medial and lateral sides of the olecranon as well as in its proximal portion, whereas the coronoid process is also fairly eroded in its anterolateral side. However, both the articular surface and the medial and lateral contours of the olecranon are very well preserved. The trochlear notch, which faces anteriorly, is short and only moderately broad (only slightly wider than the proximal portion of the shaft). It displays an hourglass-like, quite symmetrical contour in proximal view, although the lateral portion is larger than the medial one and extends posteriorly onto the shaft (thus being visible in lateral, but not in medial view). There is a moderately-developed median trochlear keel, running from the anteriormost point of the olecranon until the anterolateral portion of the coronoid process. The latter is large, concave and anteriorly-protruding, and it faces proximally, like the lateralmost portion of the distal margin of the trochlear notch, thereby indicating a spool-shaped distal humerus [4]. The radial notch, about half the size of the coronoid process, is U-shaped and faces laterally. It is situated above a relatively well-developed supinator crest (and associated fossa) for the origin of the supinator muscle, further corresponding to the beginning of the interosseous border [4]. The olecranon process is very short, slightly more proximally projecting than the trochlear notch. It is slightly posteromedially flexed (Fig. 2E). The proximal surface of the olecranon has a faint median ridge of anteroposterior direction, defining wide and shallow grooves at its both sides that correspond to weak tendon insertions for the triceps brachii muscle [5]. On the proximal shaft portion there is a well-developed crest for insertion of the brachialis muscle [4] in anteromedial position. On the medial side of the shaft, below the distal portion of the trochlear notch, there is a distinct pit for insertion of the proximalmost origin of the flexor digitorum profundus muscle and possibly for an ulnar head of the pronator teres muscle [4]. The distalmost

preserved shaft portion is anteroposteriorly higher (15.9 mm) than mediolaterally broad (12.2 mm), just like the shaft portion behind the coronoid process (anteroposterior 16.6 mm, mediolateral 12.3 mm) as well as the olecranon process (maximum anteroposterior height 21.9 mm, mediolateral breadth 15.5 mm).

References

1. Ward S, Brown B, Hill A, Kelley J, Downs W (1999) *Equatorius*: a new hominoid genus from the middle Miocene of Kenya. *Science* 285:1382-1386.
2. Sherwood RJ et al. (2002) Preliminary description of the *Equatorius africanus* partial skeleton KNM-TH 28860 from Kipsaramon, Tugen Hills, Baringo District, Kenya. *J Hum Evol* 42:63-73.
3. Aiello L, Dean CD (1990) *An Introduction to Human Evolutionary Anatomy*. Academic Press, San Diego.
4. Rose MD, Nakano Y, Ishida H (1996) *Kenyapithecus* postcranial specimens from Nachola, Kenya. *Afr Stud Monogr* Suppl 24:3-56.
5. Nakatsukasa M, Yamanaka A, Kunimatsu Y, Shimizu D, Ishida H (1998) A newly discovered *Kenyapithecus* skeleton and its implications for the evolution of positional behavior in Miocene East African hominoids. *J Hum Evol* 34:657-664.
